# Parenteral Nutrition as a Strategic Therapeutic Option for Onco-Geriatric Rehabilitation After Total Gastrectomy and Anastomotic Stenosis Complicated by an Aorto-Esophageal Fistula: A Case Report

**DOI:** 10.3390/reports9040356

**Published:** 2026-10-15

**Authors:** Houssam Eddine Belaid, João Gabriel De Carvalho, Mariangela Gagliano

**Affiliations:** 1 Private Practice, Rue Margencel 07, CH-1860 Aigle, Switzerland; 2 Department of Geriatrics, Rehabilitation and Palliative Care, Neuchâtel Hospital Network, Rue du Chasseral 20, CH-2300 La Chaux-de-Fonds, Switzerland; joao-gabriel.decarvalho@rhne.ch (J.G.D.C.); mariangela.gagliano@rhne.ch (M.G.)

**Keywords:** total gastrectomy, aorto-esophageal fistula, parenteral feeding, cancer, older patients, case report

## Abstract

**Background and Clinical Significance**: The nutritional management of onco-geriatric patients after a total gastrectomy remains a challenge, especially in the context of postoperative complications, such as stenosis of the esophagojejunal anastomosis, and in the presence of an aorto-esophageal fistula. Parenteral nutrition is a relevant therapeutic option to maintain nutritional support and enable rehabilitation when oral and enteral feeding are no longer feasible in this specific clinical context; **Case Presentation**: We report here the case of a 76-year-old patient who underwent a total gastrectomy for gastric adenocarcinoma with several dilated and stented esophagojejunal anastomoses. During the first postoperative year, the nutritional adaptation facilitated rehabilitation and helped him recover well. However, the occurrence of an aorto-esophageal fistula and its treatment with thoracic endovascular aortic repair (TEVAR) made oral and enteral feeding impossible. This was associated with persistent asthenia, physical deconditioning, sarcopenia, weight loss, exercise intolerance, and increased dependence for activities of daily living. Although parenteral nutrition allowed for nutritional stability and the beginning of rehabilitation, a suspected nosocomial infection involving the aortic endograft led to his death before his treatment was complete; **Conclusions**: This case highlights the relevance of parenteral nutrition as a therapeutic option to continue rehabilitation when enteral access is judged unacceptably risky. However, the risk of infectious complications in the context of malnutrition can compromise the prognosis, and any suspicion of infection involving prosthetic material should be documented microbiologically as completely as possible whenever compatible with the patient’s care objectives. Onco-geriatric assessment to manage older patients with esophagogastric cancer is recommended.

## 1. Introduction and Clinical Significance

According to the Global Leadership Initiative on Malnutrition (GLIM) consensus criteria, the diagnosis of malnutrition requires the combination of at least one phenotypic criterion—involuntary weight loss > 5% within the past six months (or >10% beyond six months), body mass index (BMI) < 20 kg/m^2^ in adults under 70 years (or <22 kg/m^2^ in those aged 70 years and older), or reduced muscle mass—and at least one etiologic criterion, namely, reduced food intake, impaired assimilation, or disease burden/inflammation [1,2].

Gastric cancer remains a major digestive system malignancy, with approximately 968,000 new cases and 660,000 deaths estimated worldwide in 2022. Incidence increases with age, and the proportion of patients over 75 years is steadily rising, posing complex management challenges due to underlying frailty and comorbidities [3,4].

Total gastrectomy is a major surgical procedure associated with significant postoperative morbidity, substantial weight loss, and loss of skeletal muscle mass, which are associated with impaired overall survival after gastric cancer surgery [5]. The prevalence of malnutrition in esophagogastric cancers ranges from 30 to 70%. In older adults, physiological anorexia and tumor catabolism limit the capacity to tolerate the metabolic stress of surgery, justifying systematic geriatric assessment (using the Clinical Frailty Scale, Comprehensive Geriatric Assessment, or G8 questionnaire) to estimate surgical risk, detect frailty, and adapt the rehabilitation strategy, as frailty in older patients with gastric cancer is associated with increased postoperative complications and mortality [4,5,6,7].

However, management can reach an impasse when both oral and enteral routes become high risk due to major postoperative complications, such as recurrent anastomotic strictures or aorto-esophageal fistula requiring endovascular repair (TEVAR). In this very specific clinical situation, passing a nasojejunal tube exposes patients to risks of mechanical injury or erosion in the immediate vicinity of the aortic stent, while jejunostomy may prove technically complex and high-risk due to altered anatomy and adhesions. Parenteral nutrition, then, represents a strategic therapeutic option to ensure metabolic intake and support functional rehabilitation. Nevertheless, this strategy carries risks in severely malnourished older patients, as malnutrition is independently associated with nosocomial infections and may contribute to impaired immune function and delayed wound healing [8,9].

This case report aims to illustrate the management of an elderly patient after a total gastrectomy complicated by an aorto-esophageal fistula treated with TEVAR. It highlights the educational value and challenges of a complex nutritional impasse, discusses the role and limitations of parenteral nutrition within the care pathway, and emphasizes the importance of early individualized anticipation of nutritional strategy in the onco-geriatric journey.

## 2. Case Presentation

A 76-year-old patient with no history of alcoholism or smoking was managed for adenocarcinoma of the gastric cardia. His only comorbidity was type 2 diabetes treated with oral antidiabetic agents. Preoperative geriatric assessment had not been formally performed, but his Clinical Frailty Scale (CFS) score was retrospectively estimated at 4 (vulnerable patient).

Following neoadjuvant chemotherapy, the patient underwent a total gastrectomy with lymph node dissection and mechanical esophagojejunostomy. The early postoperative course was complicated by recurrent anastomotic leaks requiring endoscopic stenting followed by endoscopic vacuum therapy (EVT/Endo-VAC). Two months after surgery, an initial 21-day rehabilitation phase with enteral nutrition via a nasojejunal tube (NJT) allowed for satisfactory functional recovery (functional independence measure (FIM) score of 6/7 for transfers and walking 60 m with a cane) and discharge home.

During the first postoperative year, the course of recovery was marked by recurrent anastomotic strictures (Figure 1) requiring iterative endoscopic dilations, as well as an episode of upper gastrointestinal bleeding from an anastomotic ulcer, treated by placing a self-expanding covered metallic stent of the LAMS type (lumen-apposing metal stent) (Figure 2). A few days later, a major complication occurred: massive gastrointestinal hemorrhage from an aorto-esophageal fistula, requiring the emergency placement of a TEVAR stent graft at a university hospital, with post-procedural imaging confirming proper stent positioning and no immediate complications (Figure 3).

In this context, enteral nutrition via an NJT was considered medically contraindicated for this patient. The esophagojejunal anastomosis was located in immediate proximity to the aortic segment treated with TEVAR, and passing an NJT through this area exposed the patient to a high risk of mechanical injury to the aortic stent (friction, migration, and perforation) as well as major infectious risk, with potential contamination of the stent and periprosthetic infection. Furthermore, the post-gastrectomy anatomy (Roux-en-Y jejunal limb, high anastomosis, and complex adhesions) made the placement of a feeding jejunostomy tube technically very difficult and associated with high surgical risk. This decision was made collegially during a multidisciplinary evaluation involving gastroenterology, digestive surgery, vascular surgery, clinical nutrition, and rehabilitation teams. Although there is no universal absolute contraindication to enteral nutrition after gastrectomy, the individual benefit/risk assessment specific to this case led to temporarily excluding enteral options and selecting parenteral nutrition as the safest and most appropriate option to ensure patient safety and overall comfort.

Upon admission to our rehabilitation unit, the patient presented significant physical deconditioning and rapid nutritional deterioration. Before the onset of the aorto-esophageal fistula, the patient’s weight was 61 kg (BMI: 18.9 kg/m^2^). One month later, involuntary weight loss of 10 kg was documented, corresponding to acute weight loss of 16.4% in 30 days, reducing weight to 51 kg and BMI to 16.2 kg/m^2^ (classifying the patient as severely malnourished according to GLIM criteria [1]).

Initial laboratory tests showed major hypoproteinemia with severely depressed serum albumin at 18 g/L (normal: 35–50 g/L) and prealbumin at 0.11 g/L (normal: 0.20–0.40 g/L), reflecting a state of severe hypercatabolism and profound protein depletion. Inflammatory markers showed elevated CRP at 88.3 mg/L.

The Subjective Global Assessment (SGA) score was class C (severe malnutrition). Sarcopenia was confirmed clinically and anthropometrically by marked muscle wasting (quadriceps, temporalis, and interosseous muscle atrophy) associated with significant loss of handgrip strength and functional decline (decreased FIM score).

Target nutritional requirements were estimated according to ESPEN recommendations at 25–30 kcal/kg/day (i.e., 1400 to 1500 kcal/day) and 1.2–1.5 g protein/kg/day (i.e., 65 to 75 g protein/day). The patient was identified as being at very high risk for refeeding syndrome. Preventive supplementation with IV thiamine (200 mg/day), vitamins B/C, and trace elements was initiated before starting parenteral nutrition (PN), administered via the pre-existing implanted port (port-a-cath).

A commercial 3-in-1 bag (providing a mixture of amino acids, glucose, and lipid emulsion) was initiated at a very gradual rate (10–15 kcal/kg/day) under daily biological monitoring (phosphatemia, potassium, magnesium, and glycemia). At steady state, PN provided 900 to 1100 kcal/day (including 45 to 55 g protein/day), covering 60 to 80% of target requirements. This strategy aimed not only at metabolic restoration but also at improving comfort, reducing fatigue, and allowing the patient to resume pleasurable oral feeding with a smooth texture. This management allowed for weight stabilization (increase to 52.5 kg at week 3) and better tolerance of physiotherapy sessions.

However, maintaining long-term central venous access in this severely malnourished and diabetic patient carried high infectious risk. The background of severe malnutrition and immunosuppression favored the occurrence of systemic complications, including pressure ulcers, diabetic decompensation, and suspected periprosthetic infection of the TEVAR aortic stent.

Following the onset of a septic state, two blood cultures drawn from the port-a-cath were positive for *Streptococcus parvulus* after 3 days of incubation. This germ, a commensal of the oro-digestive sphere, is compatible with bacterial translocation of digestive origin or catheter contamination. This documented bacteremia, associated with the clinical context (severe malnutrition, immunosuppression, presence of TEVAR vascular prosthesis, and long-term central venous access), raised suspicion of periprosthetic infection of the aortic endograft. However, in the absence of catheter tip culture, local samples, or dedicated imaging, this hypothesis could not be formally confirmed, and the exact source of the bacteremia remained undetermined.

This microbiological finding was discussed with the patient and his family, who opted for limitation of therapeutic interventions in favor of comfort measures. No additional catheter culture or further investigation was therefore performed.

Although catheter-related bacteremia represents a classic entry point, the presence of *Streptococcus parvulus* also suggests bacterial translocation of digestive origin, highlighting the multifactorial nature of this suspected infection (catheter-related, digestive translocation, or combination of both). In this context, management was redirected toward exclusive palliative comfort care until the patient’s death.

A concise chronological timeline of the main clinical, endoscopic, vascular, nutritional, and rehabilitation events is presented in Table 1.

## 3. Discussion

Malnutrition is extremely common in older adults and cancer patients, often accompanied by physiological anorexia related to aging. The GLIM criteria, developed through international consensus, define malnutrition by the combination of at least one phenotypic criterion (involuntary weight loss, low BMI, or reduced muscle mass) and at least one etiologic criterion (reduced food intake or impaired assimilation, or disease burden/inflammation) [1]. In cancer patients, the prevalence of malnutrition remains particularly high, with a recent systematic review and meta-analysis using the GLIM criteria reporting a pooled prevalence of 44% (95% CI: 36–52%) [2,6].

Total gastrectomy remains a major surgical intervention, frequently leading to iatrogenic vagotomy responsible for significant digestive functional disorders. Metabolically, major digestive surgery triggers an intense systemic stress state, characterized by an imbalance between protein synthesis and degradation, leading to a markedly negative nitrogen balance. In frail older patients, weakness of baseline physiological reserves alters the capacity to tolerate this metabolic imbalance, resulting in marked loss of muscle strength, more pronounced functional decline (decreased FIM score, limited walking distance), and significant delay in recovery of activities of daily living [4,7]. Recent data from the literature demonstrate that postoperative weight loss and skeletal muscle mass loss after gastrectomy are directly associated with decreased overall survival and increased complications, particularly in older subjects [5,7].

In our patient, the acute weight loss of 16.4% in one month, profound hypoalbuminemia (18 g/L), and severe sarcopenia observed strikingly illustrate these literature data. In our case, these major metabolic alterations were combined with complex anatomical complications, including recurrent anastomotic strictures and an aorto-esophageal fistula.

In this complex and critical context, nutritional support management required tailored adaptation. Although enteral nutrition via an NJT is generally recommended when the intestine is functional, its implementation can promote local complications when there is a wall defect or adjacent vascular prosthesis. This was a decision specific to our patient: due to the immediate anatomical proximity between the esophagojejunal anastomosis and the TEVAR aortic endograft, our multidisciplinary team estimated that passing an enteral tube presented an unacceptable risk of mechanical erosion or recurrent vascular hemorrhage. Data from the literature concerning this specific risk of erosion on TEVAR remain very limited.

Furthermore, ESPEN recommendations in oncology emphasize the importance of rigorously evaluating the benefit/risk ratio of each feeding route [2,4]. Feeding jejunostomy undoubtedly constitutes a valid long-term enteral option in many patients operated on for esophagogastric junction [2].

However, its performance in a patient with major adhesions, Roux-en-Y reconstruction, and critical tissue fragility carries non-negligible surgical risk. In our case, this option was judged too high-risk by the surgical and multidisciplinary teams, which oriented the choice toward parenteral nutrition.

Under these specific conditions, parenteral nutrition allowed us to avoid mechanical manipulations of the upper gastrointestinal tract and to reduce traumatic risk on the repaired vascular area. In accordance with ESPEN recommendations, parenteral nutrition is indicated as nutritional support when the enteral route is temporarily impossible, insufficient, or high-risk [2].

However, this decision must remain strictly individualized, balancing expected benefits (maintenance of lean mass, improved quality of life, etc.) and risks (particularly infectious, metabolic, and venous risks). The recent literature identifies malnutrition as an independent risk factor for nosocomial infections in older hospitalized patients [8,9]. This vulnerability is based on well-characterized pathophysiological mechanisms: combined alteration of cellular and humoral immunity (e.g., reduced T-lymphocyte function and decreased immunoglobulin synthesis), neutrophil dysfunction, and epithelial barrier disruption [9]. These immune deficits, aggravated by lean mass wasting and protein depletion, may contribute to delayed wound healing and an altered inflammatory response [8,9].

In our case, the occurrence of *Streptococcus parvulus* bacteremia in a severely malnourished and immunosuppressed patient with a TEVAR aortic endograft and a central venous catheter raised suspicion of periprosthetic infection. However, this infection could not be formally confirmed, and the exact source—catheter-related contamination, digestive translocation, or hematogenous infection of the prosthesis—remains undetermined. However, the genesis of this suspected infection is complex and multifactorial, involving major malnutrition, immune vulnerability, potential bacterial translocation, and the presence of long-term central venous access. No catheter tip culture was performed—due to the concerted reorientation toward exclusive palliative care focused on comfort—so the exact source of infection could not be formally confirmed.

Despite optimized parenteral nutritional support and rehabilitation efforts, the severity of initial cachexia and associated immunosuppression contributed to this pejorative outcome. This observation underscores the importance of early individualized nutritional assessment, the rapid recognition of nutritional deterioration, and the repeated reevaluation of the safest available feeding route. It also highlights the importance of multidisciplinary decision making in adapting the nutritional strategy to the patient’s overall care objectives.

## 4. Conclusions

This clinical case illustrates the complexity of nutritional management in a frail elderly patient after total gastrectomy complicated by anastomotic strictures and an aorto-esophageal fistula. It highlights the challenges encountered when conventional oral and enteral routes become very complex or scientifically risky.

This observation emphasizes the importance of early individualized nutritional assessment, the rapid recognition of nutritional deterioration, and the repeated reevaluation of the safest available feeding route. It also highlights the importance of multidisciplinary decision making in adapting the nutritional strategy to the patient’s care objectives [2,4].

Parenteral nutrition can represent a relevant therapeutic option when enteral gastrointestinal access is judged unacceptably risky, particularly in frail elderly patients [2]. However, this decision should not be dogmatic but strictly individualized, taking into account expected benefits, risks (particularly infectious), and patient preferences [2].

Additional studies would be needed to better define the indications and the optimal timing of parenteral nutrition in frail elderly patients undergoing major complicated gastrointestinal surgery.

## Figures and Tables

**Figure 1:**
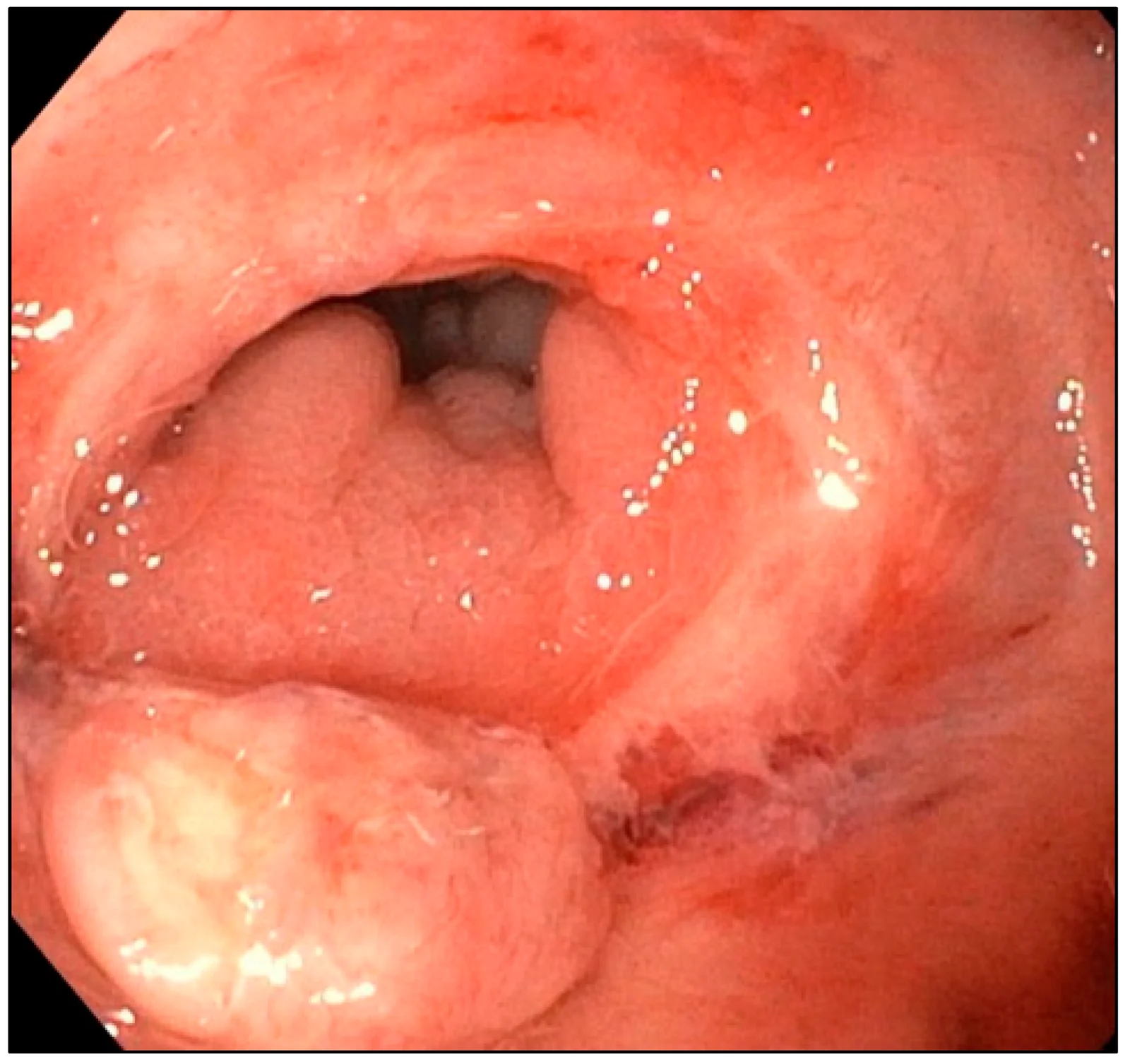
Control esophagogastric endoscopy showing recurrent anastomotic stricture. Endoscopic view of the high esophagojejunal anastomosis (located approximately 35 cm from the incisors) performed in a 76-year-old patient with a history of a total gastrectomy for adenocarcinoma, explored for severe and progressive dysphagia to solids and liquids. The examination showed fibrous and inflammatory narrowing of the anastomotic lumen, reducing the diameter to less than 5 mm and preventing passage of a standard adult endoscope. This recurrent anastomotic stricture required repeated balloon dilation sessions, complicating oral and enteral feeding and directly contributing to the patient’s acute and severe malnutrition.

**Figure 2:**
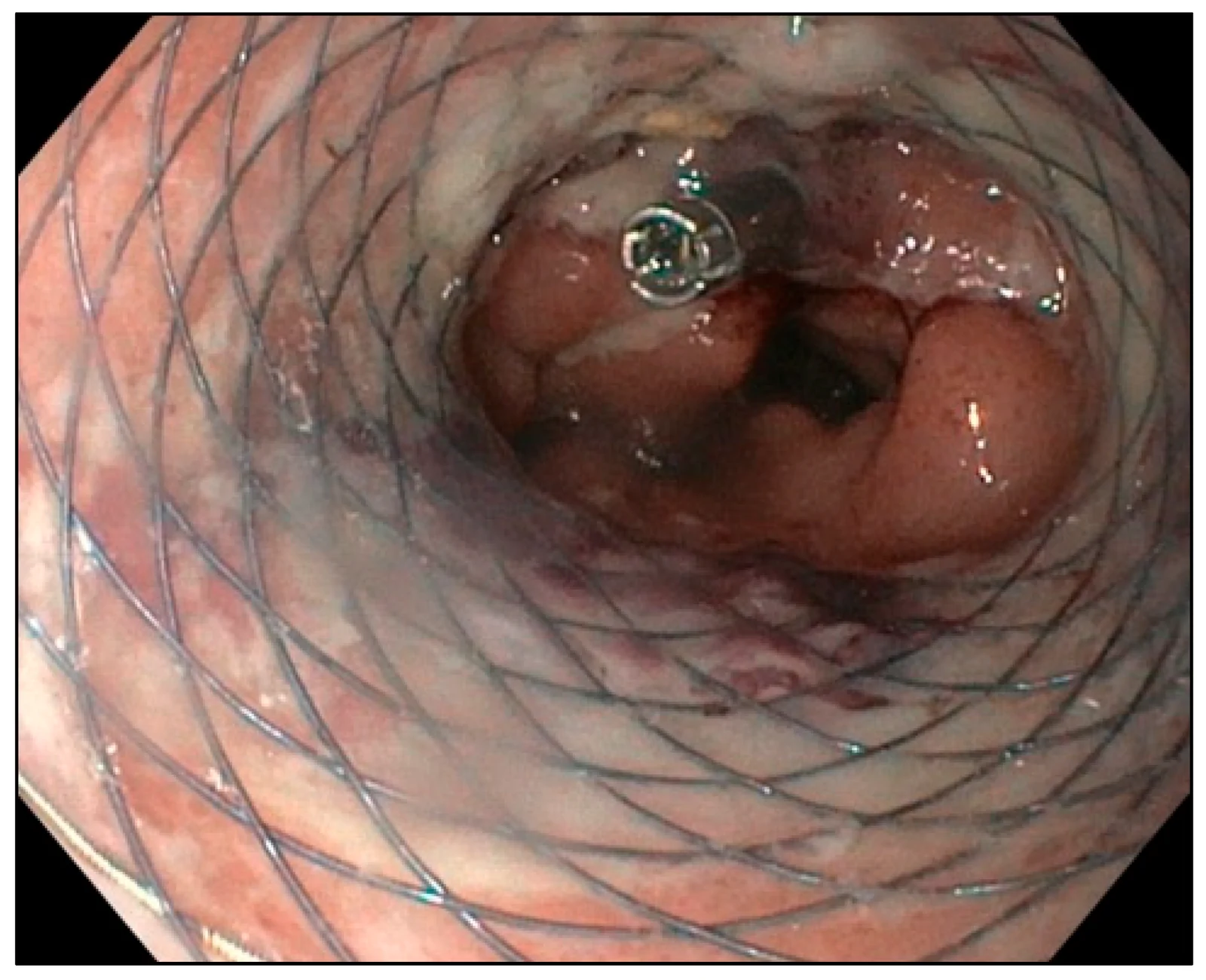
Control endoscopic evaluation of the esophagojejunal anastomosis after LAMS treatment and TEVAR stabilization. Control upper digestive endoscopy performed following stabilization of massive gastrointestinal hemorrhage secondary to an aorto-esophageal fistula complicating the anastomotic site. The examination confirms coverage of the vascular defect by the aortic endoprosthesis (TEVAR), with no active bleeding or direct mucosal erosion visible at the anastomotic stump level.

**Figure 3:**
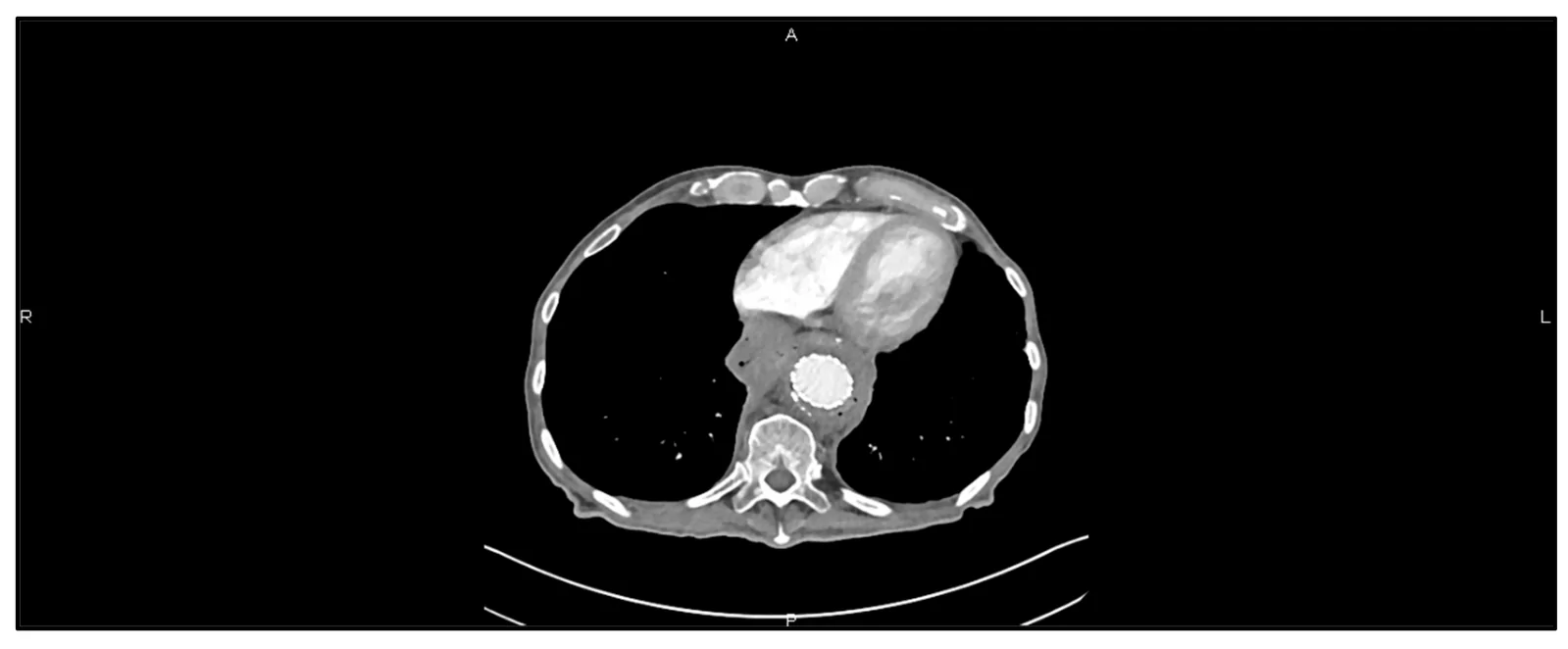
Contrast-enhanced thoracic CT scan after TEVAR placement. Axial contrast-enhanced CT (computed tomography) scan of the thorax, performed as follow-up after thoracic endovascular aortic repair (TEVAR), showing intimate contact between the descending thoracic aorta and the thickened distal esophagus, without visible fat interface. There is no esophageal opacification or demonstrable fistulous tract. This image illustrates the immediate anatomical proximity between the vascular prosthesis and the gastrointestinal tract.

**Table 1:** Chronological table summarizing the main clinical, endoscopic, vascular, nutritional, and rehabilitation events. Abbreviations: BMI, body mass index; CRP, C-reactive protein; FIM, Functional Independence Measure; GLIM, Global Leadership Initiative on Malnutrition; LAMS, lumen-apposing metal stent; NJT, nasojejunal tube; TEVAR, thoracic endovascular aortic repair.

Time Point	Main Clinical and Endoscopic Events	Vascular Events	Nutritional and Rehabilitation Assessment and Management
Preoperative period	Adenocarcinoma of the gastric cardia; neoadjuvant chemotherapy	—	No formal preoperative geriatric assessment; Clinical Frailty Scale retrospectively estimated at 4
Total gastrectomy	Total gastrectomy with lymph node dissection and mechanical esophagojejunostomy	—	Postoperative nutritional risk following major gastrointestinal surgery
Early postoperative course	Recurrent anastomotic leaks treated by endoscopic stenting followed by endoscopic vacuum therapy	—	Enteral nutrition via NJT after stabilization
Two months after surgery	Resolution of the initial postoperative complications	—	21-day rehabilitation program with enteral nutrition; FIM score 6/7 for transfers; walking 60 m with a cane; discharge home
First postoperative year	Recurrent anastomotic strictures requiring iterative balloon dilations; upper gastrointestinal bleeding from an anastomotic ulcer treated with a LAMS	—	Progressive deterioration of oral intake and increasing nutritional risk
Acute complication	Massive gastrointestinal hemorrhage secondary to an aorto-esophageal fistula	Emergency placement of a thoracic endovascular aortic repair (TEVAR) stent graft	Inability to resume oral and enteral feeding; NJT placement considered unacceptably risky in the vicinity of the aortic endograft
Rehabilitation admission	Severe physical deconditioning; sarcopenia; decreased FIM score	TEVAR aortic endograft in place	Severe malnutrition according to GLIM criteria; involuntary weight loss of 16.4% in 30 days; BMI 16.2 kg/m^2^; albumin 18 g/L; CRP 88.3 mg/L
Initiation of parenteral nutrition	—	Long-term central venous access via an implanted port	Gradual initiation of parenteral nutrition with thiamine, vitamins, and trace elements; target intake of 25–30 kcal/kg/day and 1.2–1.5 g protein/kg/day
Week 3 of parenteral nutrition	Improved tolerance of physiotherapy sessions	—	Weight stabilization at 52.5 kg; parenteral nutrition providing 900–1100 kcal/day, including 45–55 g protein/day
Late course	Septic state	Bacteremia with *Streptococcus parvulus*; suspected periprosthetic infection of the TEVAR endograft	Shared decision for exclusive palliative comfort care
Outcome	Death	—	—

## Data Availability

The data presented in this study are available on request from the corresponding author due to patient privacy.

## Abbreviations

The following abbreviations are used in this manuscript:

BMI	Body mass index
CFS	Clinical frailty scale
CGA	Comprehensive geriatric assessment
ESPEN	European society for clinical nutrition and metabolism
EVT	Endoscopic vacuum therapy
FIM	Functional independence measure
GLIM	Global leadership initiative on malnutrition
LAMS	Lumen-apposing metal stent
MIF	Functional independence measure
NJT	Naso-jejunal tube
OGD	Esophagogastroduodenoscopy
PN	Parenteral nutrition
SGA	Subjective global assessment
TEVAR	Thoracic endovascular aortic repair
